# Investigation of a Circularly Polarized Metasurface Antenna for Hybrid Wireless Applications

**DOI:** 10.3390/mi14122172

**Published:** 2023-11-29

**Authors:** Bikash Ranjan Behera, Mohammed H. Alsharif, Abu Jahid

**Affiliations:** 1 Department of Electronics and Communication Engineering, Vel Tech Rangarajan Dr. Sagunthala R&D Institute of Science and Technology (Deemed-To-Be-University), Chennai 600062, Tamil Nadu, India; drbikash@veltech.edu.in; 2 Department of Electrical Engineering, College of Electronics and Information Engineering, Sejong University, Seoul 05006, Republic of Korea; 3 School of Electrical Engineering and Computer Science, University of Ottawa, 25 Templeton St., Ottawa, ON K1N 6N5, Canada

**Keywords:** monopole antenna, polarization, artificial intelligence (AI), SADEA, intelligent metasurfaces, IoT applications, RF energy harvesting, smart sensors

## Abstract

The increasing prevalence of the Internet of Things (IoT) as the primary networking infrastructure in a future society, driven by a strong focus on sustainability and data, is noteworthy. A significant concern associated with the widespread use of Internet of Things (IoT) devices is the insufficient availability of viable strategies for effectively sustaining their power supply and ensuring their uninterrupted functionality. The ability of RF energy-harvesting systems to externally replenish batteries serves as a primary driver for the development of these technologies. To effectively mitigate concerns related to wireless technology, it is imperative to adhere strictly to the mandated limitations on electromagnetic field emissions. A TA broadband polarization-reconfigurable Y-shaped monopole antenna that is improved with a SADEA-tuned smart metasurface is one technique that has been proposed in order to accomplish this goal. A Y-shaped printed monopole antenna is first taken into consideration. To comprehend the process of polarization reconfigurability transitioning from linear to circular polarization (CP), a BAR 50-02 V RF PIN Diode is employed to shorten one of the parasitic conducting strips to the ground plane. A SADEA-driven metasurface, which utilizes the artificial intelligence-driven surrogate model-assisted differential evolution for antenna synthesis, is devised and positioned beneath the radiator to optimize performance trade-offs while increasing the antenna’s gain and bandwidth. The ultimate prototype achieves the following: an impedance bandwidth of 2.58 GHz (3.27–5.85 GHz, 48.45%); an axial bandwidth of 1.25 GHz (4.19–5.44 GHz, 25.96%); a peak gain exceeding 8.45 dBic; and when a highly efficient rectifier is integrated, the maximum RF-DC conversion efficiency of 73.82% and DC output of 5.44 V are obtained. Based on the results mentioned earlier, it is considered appropriate to supply power to intelligent sensors and reduce reliance on batteries via RF energy-harvesting mechanisms implemented in hybrid wireless applications.

## 1. Introduction

Recent years have seen a surge in the popularity of low-power embedded devices and sensors for consumer and industrial needs based on the Internet of Things (IoT), prompting researchers to focus on developing alternatives to the battery-based power supplies [1]. It unleashed the potential of ambient RF energy harvesting [2], which makes use of the electromagnetic spectrum, an interesting prospect from the perspective of the device, since it has the ability to reduce the cost with a limited need for routine maintenance. It concerns how RF energies are used in real-time scenarios. Since RF energy harvesting requires a connection to and interaction with ambient electromagnetic waves [2], RF front-ends are considered a necessary part of the process. Evaluating the performance of the components based on their circular polarization (CP) characteristics allows for improved signal matching to be accomplished [3,4]. First and foremost, it must be ensured that incoming signals from LTE, ISM, WLAN, Wi-MAX, and 5G, are correctly received regardless of the antenna’s direction. Thus, the front-end you choose is quite crucial [5,6]. Hence, we settled on the printed monopole antenna (PMAs) due to its small size, ease of analysis, good radiation efficiency, satisfactory radiation pattern, and sensitivity in the time domain.

Antenna technology has undergone rapid development in recent years. When designing effective antennas, the phenomenon of metamaterials was proposed [7], by taking into account the viewpoints of contemporary applications. It refers to something that is beyond the unusual electromagnetic (EM) properties that can be modified to take on new forms, i.e., an artificially driven engineered metasurface. In general, no material on this earth is thought to exhibit negative permeability. Every natural substance on the earth has positive permittivity, permeability, refractive index, etc. However, there is evidence of unusual negative qualities that manipulate waves and enhance the performance of antennas [8]. The evolution of the communication system has been facilitated by the emergence of several application areas due to such materials, particularly in the case of antennas [8]. As evidenced in the literature of more recent times, high-gain antennas [9] are frequently regarded as critical parts of wireless communication networks. Its importance comes from the fact that they increase signal strength, which they accomplish by minimizing interference and free space path loss (FSPL). So, the antenna performance evaluations often focus heavily on CP antenna gain. The gain has been enhanced in this article using MS approaches [9]. However, improving impedance bandwidth and CP characteristics (CP bandwidth, CP gain, and antenna efficiency) has not been investigated w.r.t. performance trade-offs [10,11,12,13,14,15,16,17,18,19,20,21,22,23,24,25,26,27,28,29,30,31]. That is why prospective applications call for a single antenna element with certain features, most notably the ability to provide enhanced CP. Vias [10], non-metasurfaces [11,12,13,14,15], and electromagnetic metasurfaces [16,17,18,19,20,21,22,23,24,25,26,27,28,29,30,31] are a few methods that have been used previously for the implementation of enhancing the antenna’s performance. So, acquiring a broadband CP is one of the requirements for a successful polarization system, targeting IoT. The works reported in [10,11,12,13,14,15,16,17,18,19,20,21,22,23,24,25,26,27,28,29] are needed to pursue a surrogate model-assisted differential evolution for antenna synthesis, that is, a SADEA-driven smart metasurface, for attaining better outcomes.

**Figure 1:**
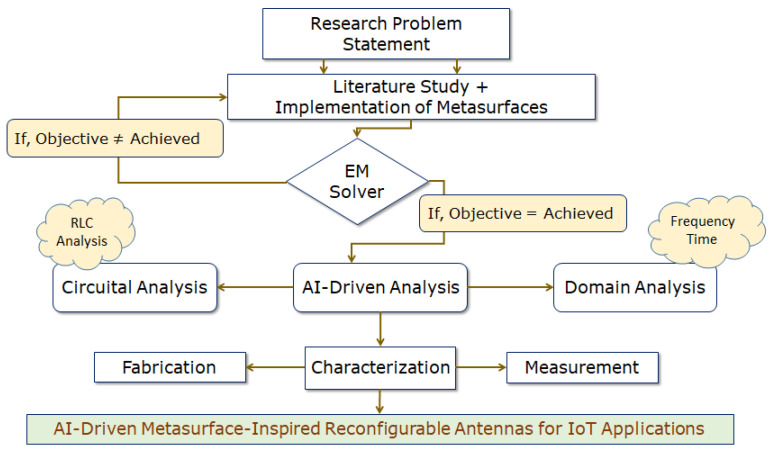
Flowchart explaining the workflow of the proposed work reported for the IoT application.

In this paper, we look at whether it is possible to design a reconfigurable, broadband, printed monopole antenna by using the surrogate model-assisted differential evolution for antenna synthesis, that is, a SADEA-driven metasurface. Here, a BAR 50-02 V RF PIN Diode is used to short partial ground to one of the parasitic conducting strips (i.e., PCS_L_) and create a broadside directional pattern while also incorporating the metasurface of evenly and symmetrically spaced unit cells. Thus, the antenna’s gain was increased over its operating frequency range, and its impedance and axial bandwidths were also enhanced, contributing to stable antenna performance [32]. In a concurrent way, all three aspects of CP analysis [33] were addressed; (a) electric field distribution, (b) surface current distribution, and (c) far-field radiation are modelled and interpreted in a comprehensive analogy. Ahead of it, (d) amplitude-phase responses are also examined over the desired operating frequency band. So, the fact that CP is provided in the form of distinctive gain-bandwidth product (GBR) connections aids in persuading the reader that they exist. Towards these ends, the authors followed the DAVI principle: Designing, Analyzing, Validating, and Implementing. A flowchart showing the boarding process involved during this present work is shown in Figure 1.

## 2. Antenna Design

**Figure 2:**
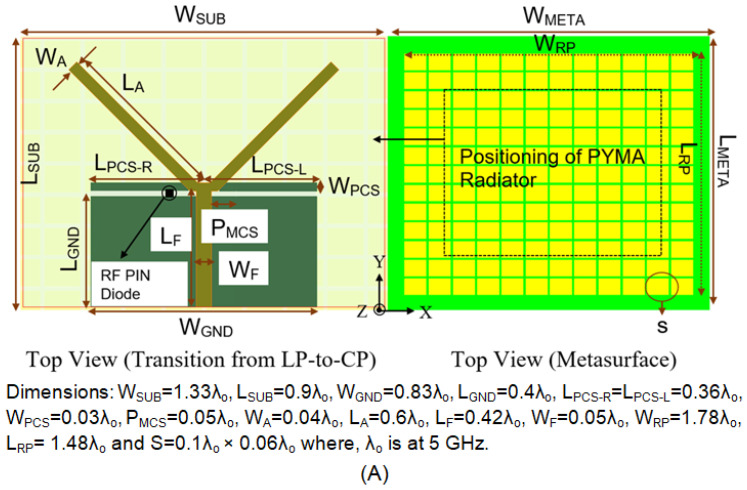

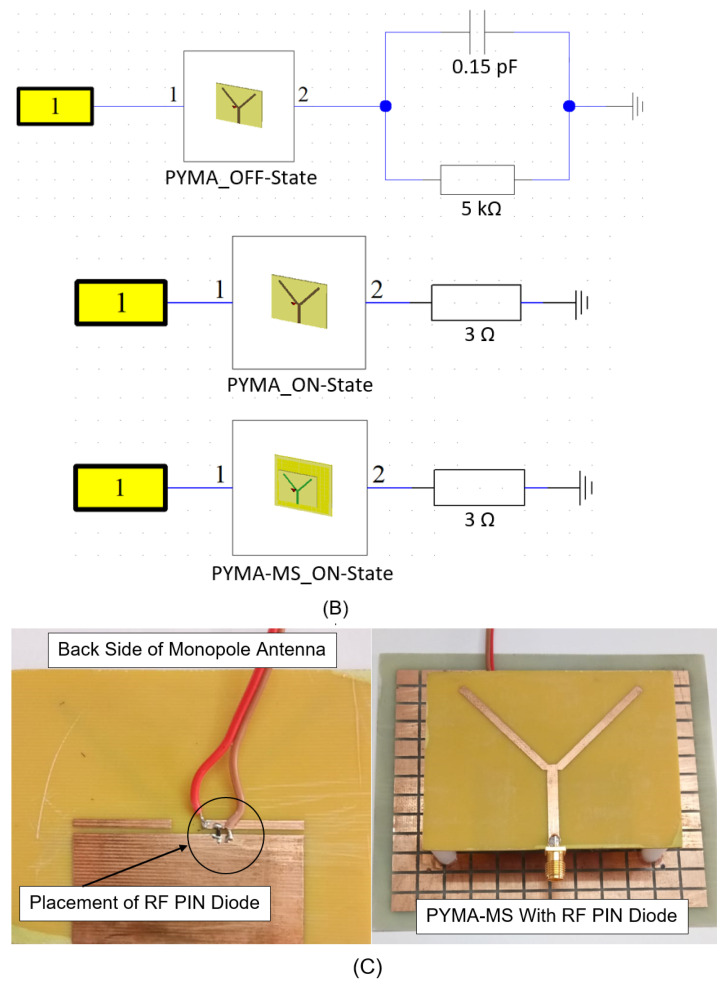
Design methodology of the proposed antenna: (**A**) schematic, (**B**) biasing, and (**C**) prototype.

**Figure 3:**
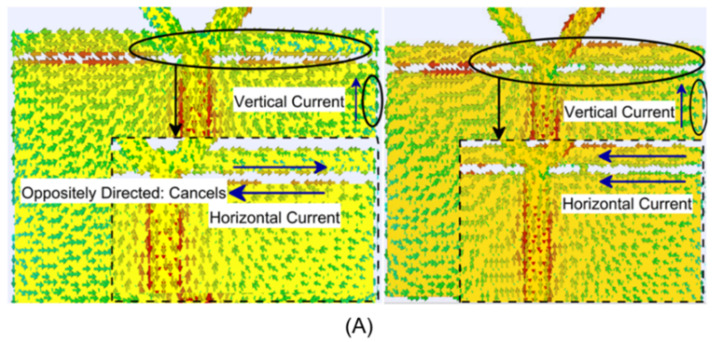

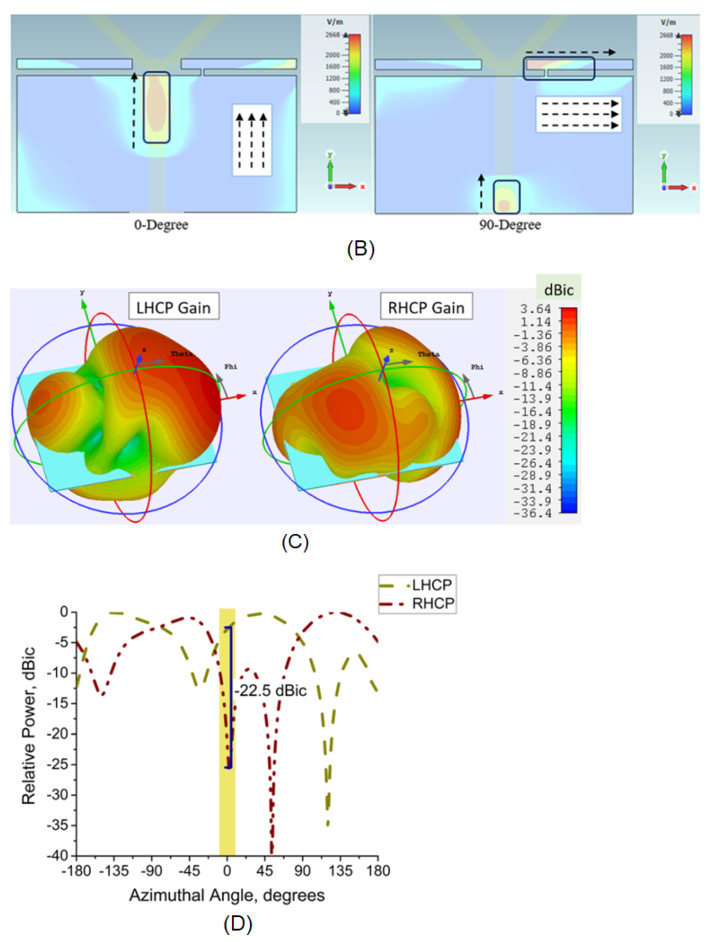
CP at 5 GHz: (**A**) 1st method; (**B**) 2nd method; and (**C**,**D**) 3rd method (3 fundamental aspects of CP analysis).

**Figure 4:**
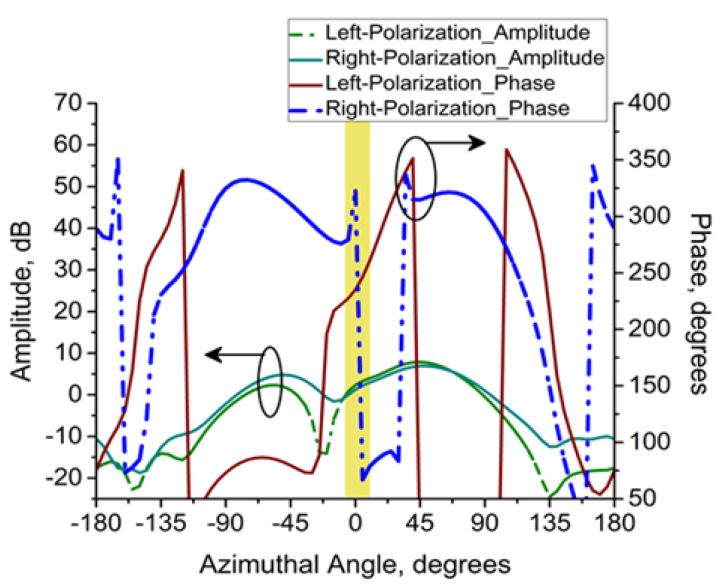
CP at 5 GHz (cont.): Amplitude and phase responses.

**Figure 5:**
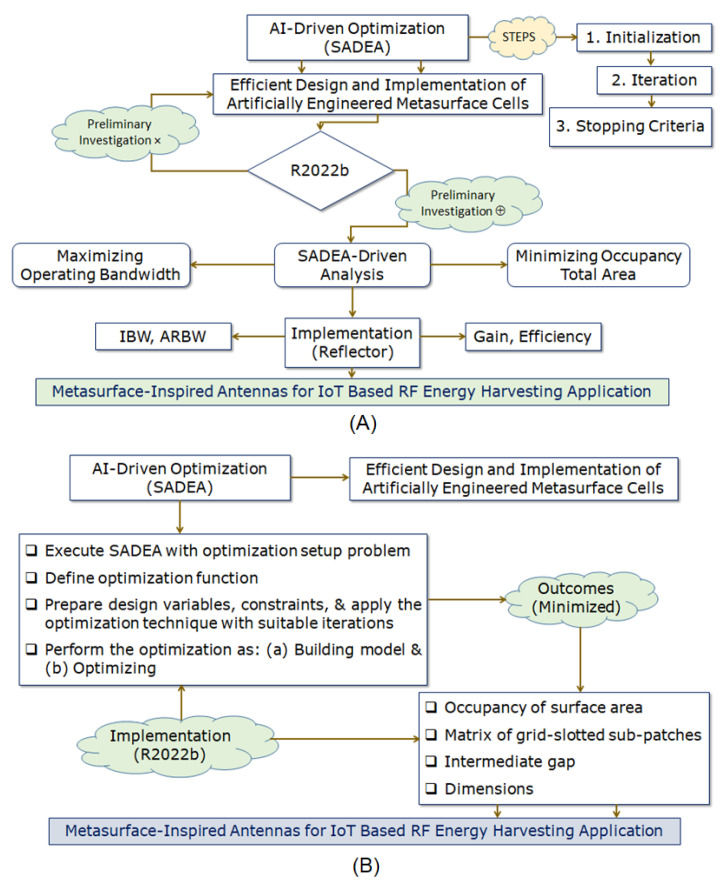
(**A**) SADEA: Process and (**B**) SADEA: Implementation using MATLAB 2022b platform.

**Figure 6:**
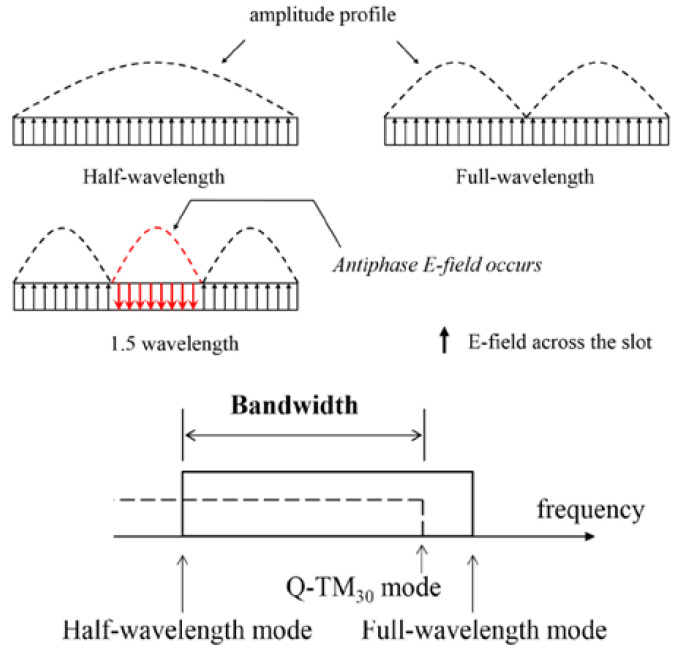
Generation of higher-order modes of artificially engineered metasurface (RMS).

**Figure 7:**
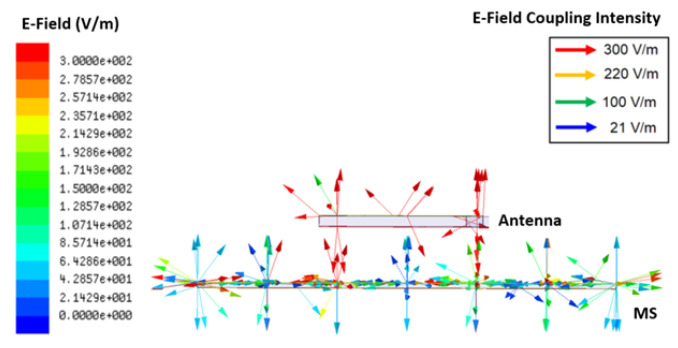
Coupling phenomena of artificially engineered metasurface (RMS).

**Figure 8:**
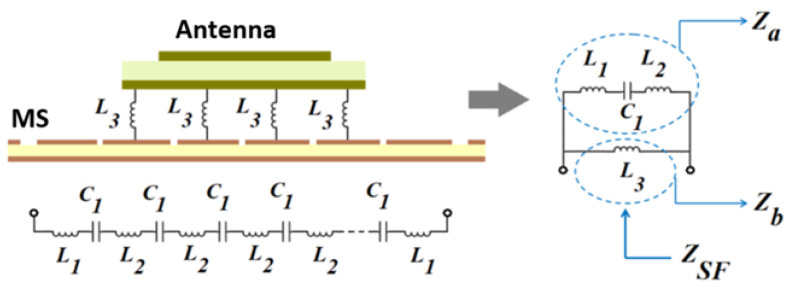
Circuit representation of artificially engineered metasurface (RMS).

**Figure 9:**
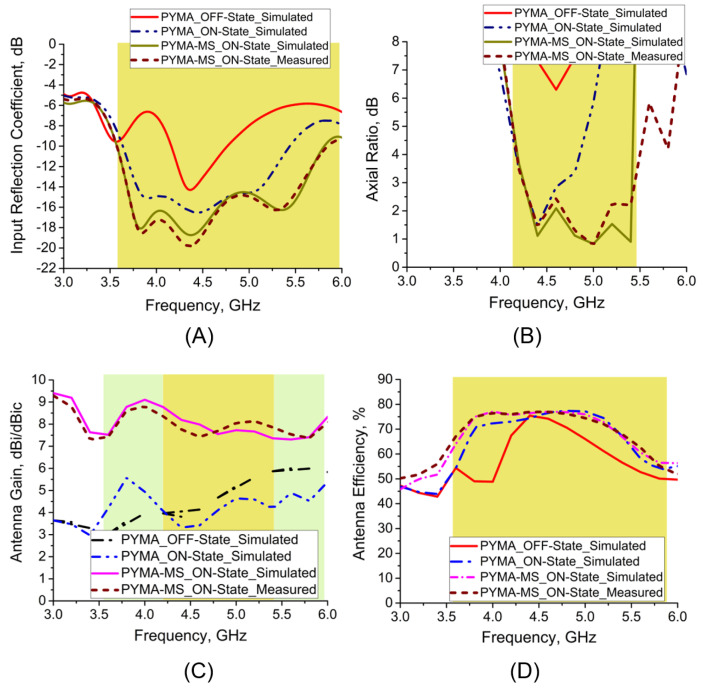
Antenna performances: (**A**) IBW, (**B**) ARBW, (**C**) antenna gain, and (**D**) antenna efficiency.

**Figure 10:**
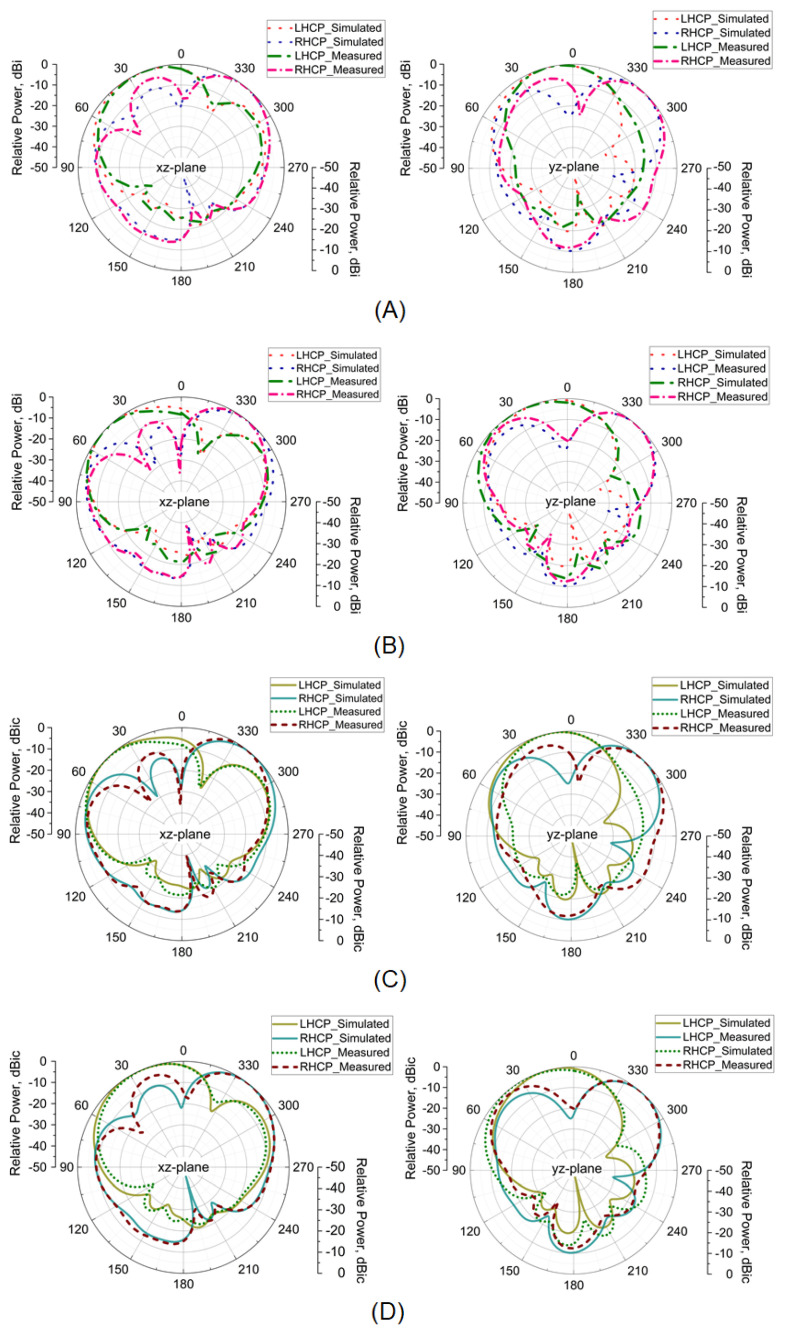
Radiation patterns: (**A**) 4.5 GHz (OFF-state), (**B**) 5 GHz (OFF-state), (**C**) 4.5 GHz (ON-state), and (**D**) 5 GHz (ON-state).

**Figure 11:**
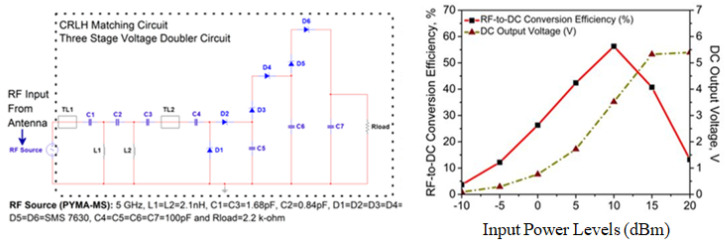
Rectifier circuit-I with its outcomes for the proposed AI-tuned CP metasurface antenna.

**Figure 12:**
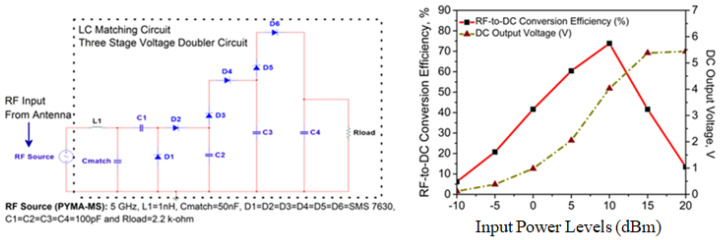
Rectifier circuit-II with its outcomes for the proposed AI-tuned CP metasurface antenna.

The proposed antenna is fabricated on an FR-4 substrate by incorporating the twin parasitic conducting strips (PCSs) 1.16 mm from the upper edges of the partial ground plane, 21.6 mm in length and 1.84 mm in width, where the authors extended the ground plane following the performance trade-offs [33]. The communication in between the partial ground plane and one of the parasitic conducting strips (PCS_L_) is crucial for CP features, achieved by shorting the partial ground and PCS_L_ using a BAR 50-02 V PIN Diode. A 50-Ω microstrip feedline is used for the input excitation. The proposed antenna is presented in Figure 2, with state-of-the-art results in Figure 3, Figure 4, Figure 5, Figure 6, Figure 7, Figure 8, Figure 9, Figure 10, Figure 11 and Figure 12. During the OFF-state, it attains an impedance bandwidth (IBW) of 620 MHz with no CP bandwidth, whereas, in the ON-state, it attains an impedance bandwidth (IBW) of 2.11 GHz with a CP bandwidth (ARBW) of 460 MHz. The average antenna gain in both states of operation is around 3.2 dBi. With the implementation of a SADEA-driven metasurface reflector, it is possible to obtain broadband CP, CP gain of >8.2 dBic, antenna efficiency of >75%, and directional pattern.

## 3. CP Mechanism: CEM Approach

Figure 3A–D and Figure 4 show the different responses to CP analysis [33] persuaded at the 5 GHz band. Here, the surface current distribution is the CP analysis’s initial and most fundamental aspect. Thus, in the OFF-state, parasitic conducting strips (PCSs) play no role in the non-active device without a connection to the partial ground plane. It correlates to the fact that the surface currents caused by the horizontal margins of the partial ground plane flow in the opposite direction. Due to mutual cancellation, only vertical surface currents on the printed monopole arm remain. This results in a wave with linear polarization (LP). When one of the parasitic conducting strips (PCS_L_) is connected to the partial ground plane by the BAR50-02 V RF PIN Diode, the surface currents on the PCS_L_ and the partial ground plane are rearranged so that the resultant currents on the upper edge of the PCS_L_ and the lower edge of the partial ground plane do appear in the same direction. So, this accounts for horizontal surface currents while the ON-state is active. As a result of it, the viability of CP features depends on the presence of horizontal and vertical components, which can also be referred to as horizontal and vertical currents, making for the viability of CP.

Analysis of the CP can also be performed by looking out at the electric field distribution as the second primitive method. Therefore, the CP reconfigurable antenna realizes LHCP in the +*z* direction (i.e., outward in nature) when PCS_L_ is coupled to the partial ground plane with the BAR50-02 V RF PIN Diode. At 5 GHz, the rotation of electric field vectors goes from anti-clockwise to clockwise, which causes the phase to shift from 0° to 90°. Thus, the formation of LHCP (left-handed circular polarization) is verified through the presence of an orthogonal change in the electric field pattern.

The normalized radiation pattern constitutes the third and final primitive method for CP analysis. Here, a correlation is found w.r.t. the relative power at 5 GHz. The proposed antenna possesses exceptional LHCP properties. Techniques like the first, second, and third have been used to investigate the CP’s explanation reported here. The amplitude and phase responses are presented in a much better manner so that the CP features can be understood.

## 4. AI-Tuned Design and Implementation of Metasurface (RMS)

Antenna Toolbox^TM^ in MATLAB provides users access to the various functions and applications that may be used to design, analyze, and visualize antenna elements. These elements include the metasurface unit cells, which are an essential component of artificially engineered metasurfaces [32]. The question is whether it can be generated utilizing parametrically defined elements, arbitrary planar structures, or isolated three-dimensional forms. Because it uses an EM solver, it is just as competent as the methods of moments of computing all of the desired characteristics to carry out the final verification. The toolbox allows manually optimizing the antenna designs [34] so that they can be optimized in the most effective way possible. An approach for designing an antenna that is powered by artificial intelligence (AI) is known as SADEA, which stands for the surrogate model-assisted differential evolution for antenna synthesis. SADEA is an acronym for this method. It is based on the theories of machine learning [34] and evolutionary computation.

The global optimization is performed with the help of SADEA, and statistical learning techniques are used to build a surrogate model. Thus, it is of the utmost importance, in the context of the surrogate model-assisted optimization approach, that the process by which the surrogate modeling and optimization are made to cooperate successfully is the surrogate modeling and the optimization [35]. In addition to that, some ideas taken from an evolutionary search framework that takes into account surrogate models have been incorporated into SADEA [36,37]. Both the search engine that SADEA uses, called differential evolution (DE), and the machine learning technique that is used for surrogate modeling, known as a Gaussian process (GP), are described in [38,39]. So, in the case of metasurface-inspired antenna design, SADEA optimization appears to be utilized only in extremely unusual circumstances and is reported as very rare in the literature to design artificially engineered metasurfaces. The MATLAB R2022b platform initiates the process of designing an RMS layer to improve the antenna with consideration of the IoT application perspective, which can be seen in Figure 5.

By using the AI-driven SADEA method, the optimization goal is to achieve improvement in the outcomes: (a) maximizing operating bandwidth with center frequency at 5 GHz and (b) minimizing the area of occupation for the case of the designed metasurface layer (RMS). Thus, a critical observation is made during this analysis, as RMS plays a vital role in maintaining the antenna’s performance, considering the trade-offs from an application perspective. Figure 9 shows the SADEA-optimized outcomes (simulation), along with its interpretation of the measured outcomes. So, in continuation with the motive of attaining improved outcomes, the execution is performed for the RMS reflector (with the optimized dimensions for the size of grid-slotted sub-patch cells, intermediate gap, and overall size of the RMS layer) at the height of 0.33λ_∘_ below the Y-shaped monopole antenna to obtain good operating bandwidth, a high CP antenna gain, and enhanced directional features.

Here, the reflector layer is made in the form of RMS, which has a surface area of 1.78λ_∘_× 1.48λ_∘_, shown in Figure 2. It consists of grid-slotted sub-patches of the 12 × 12 cells, as each cell is 0.1λ_∘_× 0.06λ_∘_ and has an intermediate gap of 0.016λ_∘_. Further, the sub-patches are well positioned on a rectangular-shaped PEC body having the dimensions of 2λ_∘_× 1.65λ_∘_× 0.02λ_∘_ (λ_∘_ = 5 GHz). They are merged to form a rectangular-shaped metasurface reflector, which is loaded with a polarization-reconfigurable monopole antenna.

Carrying out the analysis further, in Equation (1), other parameters, like the thickness of the substrate (h_sub_), the relative permittivity of the substrate (ϵ_r_), and λ_∘_, each have a role in determining the effective gap (h_air-gap_) in between the radiator and AI-tuned RMS. The theoretical h_air-gap_ is 21.85 mm, but the simulated h_air-gap_ measures 20 mm. This study also shows the final mathematical formula, which is then used to look at how to place reflectors in relation to the radiator, taking into account how often it works and not its structure. Its building blocks are a gridlike pattern of tiny elements printed on a grounded slab, with or without shorting vias. Through this analytical approach, one can easily approximate the h_air-gap_:

(1) $$h_{air-gap}=0.42\lambda_{o}-h_{sub}\sqrt{\varepsilon_{r}}$$

The average CP gain increases by a factor of 3.65 times, from 2.35 dBic to 8.58 dBic. More importantly, the antenna’s IBW increases by a factor of 1.08 times, from 2.11 GHz to 2.28 GHz, and its ARBW increases by a factor of 2.7 times, from 460 MHz to 1.25 GHz. This is motivated by geometric understanding, as the development of such antenna performances aims to realize the performance trade-offs. So, the proposed RMS unit cells are shown to concentrate radiation fields for the occurrence of quasi-TM_30_ modes (i.e., the transformation of TM_10_ to TM_30_ modes) using the finite integral technique (FIT), resulting in a more uniform E-field distribution and ultimately, better impedance performance over a wide frequency range. Higher modes [40] are shown to be caused by RMS unit cells in the form of grid-slotted sub-patches in Figure 6. Here, the impedance matching is improved in the antenna design by minding the gaps between sub-patches where radiation can behave differently [41,42,43,44,45], which holds the capacity of manipulating these EM waves towards constructive interference.

The coupling phenomena and circuit model of the proposed antenna are depicted in Figure 7 and Figure 8. The RMS layer is made up of a series of inductive and capacitive layers that are stacked one on top of the other. Layer *L*_1_ serves as the RMS external radiating border and is connected to the inductive unit cells that are located on layer *L*_2_, which capacitive layer *C*_1_ then separates. Following it, the inductance *L*_3_ layer establishes connections with the ground and the RMS layers, because the overall inductances, which include the dielectric substrate of the proposed antenna as well as the air substrate that is located in between the dielectric substrate of the antenna and the metallic RMS component, are theoretically represented by *L*. To simplify the analysis, a generalized equivalent circuit will be stated as parallel impedances (*Z*_a_||*Z*_b_). The equivalent surface impedance is calculated, and its frequency dependency w.r.t. RLC is shown in Equations (2) and (3):

(2) $$\eta_{eq}=\frac{j\omega L_{3}\left(1-\omega^{2}L_{1}C_{1}-\omega^{2}L_{2}C_{1}\right)}{1-\omega^{2}L_{1}C_{1}-\omega^{2}L_{2}C_{1}-\omega^{2}L_{3}C_{1}}$$

(3) $$f_{R}=\frac{1}{2\pi \sqrt{\left(L_{1}+L_{2}+L_{3}\right)}C_{1}}$$

## 5. CP Mechanism: GBR Approach

To further evaluate the CP, the gain-bandwidth product relationship, Equation (4) states:

(4) $$C_{criteria}=F(BW_{3-dB},G_{3-dB})$$

Then,

(5) $$C_{criteria}=BW_{3-dB}\times G_{3-dB}$$

Further,

(6) $$C_{criteria}=\frac{BW_{3-dB}\times G_{3-dB}}{100}$$

Equation (6) can be re-written as follows:

(7) $$C_{criteria-1}=\frac{BW_{3-dB}\times G_{3-dB(avg)}}{100}$$

(8) $$C_{criteria-2}=\frac{BW_{3-dB}\times G_{3-dB(max)}}{100}$$

(9) $$C_{criteria-3}=\frac{BW_{3-dB}\times G_{3-dB(min)}}{100}$$

(10) $$C_{criteria-4}=\frac{BW_{3-dB}\times G_{3-dB(peak)}}{100}$$

The limitations of traditional comparison methods that were discovered earlier are addressed in a major way by *C*_criteria-1_-*C*_criteria-4_. In traditional comparison methods, these CP antennas are practically compared to only one feature. Equation (6) is the generalized equation, often known as the gain-bandwidth product (GBR), for evaluating the CP antennas. So, this particular study was presented in the literature for the very first time as a research evaluation. Comparative research with work already published in [10,11,12,13,14,15,16,17,18,19,20,21,22,23,24,25,26,27,28,29] is included in Table 1 to bolster the study by providing further evidence in this context. By including major evaluative components such as the CP bandwidth and CP gain, this article offers a novel perspective on the investigation of CP that is both informative and eye-opening. Further, the examination of CP features that were covered before is implemented in every case, even though the antenna shape/frequency at which it works differ from one another.

## 6. Measurement

In Figure 2C, the fabricated prototype of the proposed polarization-reconfigurable monopole antenna inspired by RMS is shown. Figure 9A,B shows that the simulated antenna measured impedance bandwidths (IBWs) as follows: 3.59–5.78 GHz, 2.19 GHz, 46.71% and 3.57–5.84 GHz, 2.27 GHz, 48.45%. The simulated and measured axial bandwidths (ARBWs) are as follows: 4.22–5.28 GHz, 1.06 GHz, 23.31% and 4.19–5.44 GHz, 1.25 GHz, 25.96%, respectively. Similarly, the average simulated and measured CP antenna gain lies between 7.5 and 9.35 dBic, with the average antenna efficiency of >75% in their operating bands, shown in Figure 9C,D. Furthermore, the radiation patterns at 4.5 and 5 GHz are presented in Figure 10A–D.

5G [2] is widely regarded as an advanced wireless technology that offers significant economic potential for facilitating the translation of ideas into practical solutions, thereby overcoming obstacles and fostering the creation of diverse strategies. However, the path loss associated can be detrimental to the overall system performance. So, a plausible solution to mitigate this will be to use a highly directive, efficient antenna with enhanced performance capabilities. Thus, to operate in the 5G band, these antennas must also have wide operating bandwidth (>1 GHz) covering the entire allotted band along with other performance trade-offs [32], reported here as a widespread solution [46,47,48,49,50,51].

## 7. RF Energy Harvesting: Simulation Perspective from IoT Application

The design and implementation of a front-end that operates with enhanced performances, due to the incorporation of RMS, and embedded with the multi-stage rectifier circuit system (GVDs) are said to be important to satisfy these types of trade-offs [2].

The overall results that are displayed in Figure 11 and Figure 12 are appropriate for powering these sensors that can be found in low-power devices (such as wearables, medical, and healthcare plug-based kits), which need a consistent DC output voltage of 2.4–5.5 V to function properly. In a nutshell, the application of this particular technology will progressively become an essential component in the process of developing effective systems. Despite the various challenges it faces, once these obstacles are overcome, it will usher in an age of clean, green, and sustainable energy suitable for 5G and 6G applications [52,53].

To briefly summarize and assess the RF energy-harvesting capabilities, it is combined with the GVD rectifier circuit. Within this circuit, the theoretical analogies are supplied for the RF-DC power conversion efficiency (η_∘_, %) and DC output voltage (V_out_, V). The three-stage Greinacher Voltage Doubler, complete with a CRLH- and LC-matching rectifier circuit, has been designed and incorporated into the proposed antenna. It is tested for input power levels (*P*_in_) between 0 and +20 dBm, where η_∘_> 60% and V_out_> 2.1 V at 5 dBm, when it is simulated at the ADS platform with a load resistance (*R*_load_) of 2.2 kΩ. Table 2 contains an in-depth performance study, compared with some of the reported works in [46,47,48,49,50,51]. Finally, the η_∘_ is calculated by using Equation (11), and multi-stage GVD configuration is explained based on Equations (12) and (13).

(11) $$\eta_{0}\left(\%\right)=\frac{P_{load}}{P_{incident}}=\frac{V_{out}^{2}}{P_{in}\times R_{load}}$$

Theoretical insights into the proposed rectifier model are also studied prior to the simulation in the ADS environment with the LSSP scenario. So, in this case, each stage with its own GVD configuration is regarded as a single battery with an open circuit output voltage (V_o.c._), internal resistance (*R*_int_), and load resistance (*R*_load_). The DC output voltage is given by:

(12) $$V_{out}=\frac{V_{o.c.}}{R_{int}+R_{load}}\times R_{load}$$

For *n* number of stages in series and connected to *R*_load_, then V_out_ is represented as:

(13) $$V_{out}=\frac{nV_{o.c.}}{nR_{int}+R_{load}}\times R_{load}$$

So, it is observed in our analysis that the total number of stages in the system has a substantial impact on the output voltage. The utilization of a partial ground plane in the proposed antenna was what resulted in the maximization of the captured energy, which can energize the sensors used in IoT applications. This realization of a higher amount of DC output voltage can be attributed to utilizing a partial ground plane concept. In the continuation of our inquiry, we have found that the application of RMS improved the gain of the RF front-end. When evaluating the effectiveness of a rectenna model in an RF energy harvesting system, one of the most important metrics to look at is the amount of power that is received by the antenna. This is because the power received by the antenna is directly proportional to the model’s performance. Given the particular parameters of operating frequency and the availability of RF signals, the only feasible option to maximize the results of RF energy-harvesting is to increase the 3 dB gain of the CP-printed monopole antenna.

## 8. Implementation Perspective: A Quick Overview

- This simple antenna employs the monopole and parasitic conducting strip configurations. Because the polarization pattern is meant to look like a CP, there is no need for electromagnetic circuits for it to work well.
- The incorporation of an RF PIN Diode (BAR 50-02V from Infineon Technologies) in a unique manner avoids using additional lumped parts due to planar configuration and less complex antenna design to attain polarization reconfigurability (i.e., LP to CP).
- CP is examined using a novel gain-bandwidth product. Also, four methodologies are shown to highlight the significance of CP feature analysis at the operating bandwidth.
- With the addition of a SADEA-driven metasurface (RMS) reflector in a single layer, there is a significant improvement in impedance and axial ratio bandwidth responses (broadband characteristics). An enhanced CP antenna accompanies this improvement gain > 8.45 dBic, which provides a superior performance in comparison to [10,11,12,13,14,15,16,17,18,19,20,21,22,23,24,25,26,27,28,29], from the perspective of IoT-inspired RF energy harvesting, aiming towards energy-efficient communication and computing technologies.
- Here, the η_∘_ and V_out_ are computed by ADS. The proposed SADEA-driven metasurface antenna is tested with the CRLH- and LC-based Grienacher voltage doubler circuits (GVD). The antenna system has CRLH- and LC-based Grienacher voltage doubler circuits (GVD) built into it so that the potential utility of the system can be tested out in relation to an application that uses RF energy harvesting, which presents a more effective method towards implementing rectifiers than those stated in [46,47,48,49,50,51]. Also, in this case, the maximum V_out_ that can be attained is 5.39 V (for I) and 5.44 V (for II), and the maximum η_∘_ that can be attained is 56.28% (for I) and 73.82% (for II), respectively. A comparative study in this regard is already given in Table 2.

## 9. AI-Enabled IoT Applications towards Smart Living: A Future Research Direction

Self-sustainable smart sensors through the RF energy-harvesting mechanisms can be a path-breaking innovation for elderly people, as they require less maintenance and supervision. One of the major concerns in the utilization of these sensors by elderly people is the way they are powered; there is always an increasing demand for these devices to be automated, especially for those who have memory problems. So, there exists significant research need for automating the battery charging process in these wearables, and on the other hand, the continuous availability of alternate energy sources from the open space, rendering a viable option for harnessing RF energy. In recent years the concept of energy harvesting has emerged as a promising solution, which, if incorporated into these low-powered smart devices, has a promising scope [52,53,54].

## 10. Conclusions

This work examines a SADEA-driven metasurface-inspired printed monopole antenna. It offers a broadened IBW and ARBW with a measured CP gain of >8.45 dBic and antenna efficiency of >75% in its operating bands. Along with their physical insights, intuition about the CP is also presented with three different aspects based on computational electromagnetism. A complete design is given for the wide-scale solution to a problem that the printed monopole antennas have, mainly when their CP characteristics are analyzed, as the unique gain-bandwidth product relationship, which is rarely seen in the literature, targeting IoT-inspired applications. As a result, the work that is being reported here combines electromagnetics with artificial intelligence-driven SADEA to achieve better results from an application perspective regarding CP characteristics. This is carried out with the goal of satisfying the primary needs of the human race, which are aimed at powering the sensors (known as RF energy harvesting).

## Figures and Tables

**Table 1:** Comparison of the performances of the proposed antenna with the ones reported in [10,11,12,13,14,15,16,17,18,19,20,21,22,23,24,25,26,27,28,29].

Ref.	IBW	ARBW	CP Gain	Peak Gain	*C*_criteria-1_	*C*_criteria-4_
[10]	7.29%	5.5%	6.5 dBic	7.1 dBic	0.35	0.39
[11]	28.6%	14.2%	4.4 dBic	4.4 dBic	0.62	0.62
[12]	31.6%	20.8%	6.9 dBic	6.9 dBic	1.43	1.43
[13]	35.6%	16.3%	5.5 dBic	5.5 dBic	0.89	0.89
[14]	29.6%	20.7%	7.44 dBic	7.44 dBic	1.54	1.54
[15]	12.68%	12.68%	6.8 dBic	6.8 dBic	0.86	0.86
[16]	20.89%	11.4%	5.2 dBic	7.5 dBic	0.59	0.85
[17]	19.6%	15.8%	4.25 dBic	6.5 dBic	0.67	1.02
[18]	33.7%	16.5%	5.8 dBic	5.9 dBic	0.95	0.97
[19]	17.8%	3.6%	7.8 dBic	8.2 dBic	0.28	0.29
[20]	27.5%	7.8%	6.1 dBic	5.8 dBic	0.47	0.45
[21]	20%	20%	6.1 dBic	6.6 dBic	1.22	1.32
[22]	8.4%	4%	6.1 dBic	6.95 dBic	0.24	0.27
[23]	31.8%	20.4%	7.47 dBic	8.05 dBic	1.52	1.64
[24]	15.1%	12.4%	6.15 dBic	6.5 dBic	0.76	0.81
[25]	27%	2.5%	3.02 dBic	3.02 dBic	0.07	0.07
[26]	1.55%	1.05%	7.8 dBic	8.2 dBic	0.07	0.08
[27]	25.6%	25.6%	7.3 dBic	6.25 dBic	1.6	1.86
[28]	23%	14%	——–	———	——–	——–
[29]	7%	8.3%	——–	———	——–	——–
**Work**	**48.45%**	**25.96%**	**8.45 dBic**	**9.15 dBic**	**2.19**	**2.37**

**Table 2:** Comparison of RF energy-harvesting features with the reported ones in [46,47,48,49,50,51].

Ref.	Gain	*P*_in_	η _∘_	V_out_
[46]	5.85 dBic (CP)	5 dBm	50%	——
[47]	6.9 dBi (LP)	5 dBm	——	0.1 V
[48]	7.3 dBi (LP)	5 dBm	14%	1.1 V
[49]	5.01 dBic (CP)	5 dBm	43%	1.16 V
[50]	5.5 dBi (LP)	5 dBm	5%	0.2 V
[51]	2.6 dBi (LP)	5 dBm	55%	——
Present Work	>8.45 dBic (CP)	5 dBm	43% (I)	1.7 V (I)
	>8.45 dBic (CP)	5 dBm	61% (II)	2.1 V (II)

## Data Availability

Data are contained within the article.
